# Increased medial talar tilt may incite ankle pain and predispose ankle osteoarthritis after correction of severity of knee varus deformity among patients undergoing bilateral total knee arthroplasty: a prospective observation

**DOI:** 10.1186/s43019-024-00212-x

**Published:** 2024-01-24

**Authors:** Arghya Kundu Choudhury, Shivam Bansal, J. Pranav, Balgovind S. Raja, Tushar Gupta, Souvik Paul, Kshitij Gupta, Roop Bhushan Kalia

**Affiliations:** 1 Centre of Robotics and Joint Replacement Surgery, Sarvodaya Hospital and Research Centre, Faridabad, India; 2 Department of Orthopaedics, All India Institute of Medical Sciences, Rishikesh, India; 3 Department of Orthopaedics, All India Institute of Medical Sciences, Patna, India; 4 AMRI hospital, Department of Orthopaedics, Mukundapur, Kolkata, West Bengal India

**Keywords:** Ankle pain, Total knee arthroplasty, Total knee replacement, Varus talar tilt, Varus incongruency, Post-TKA, Varus knee, Severe varus knee osteoarthritis

## Abstract

**Purpose:**

Patients with varus knee osteoarthritis usually compensate at the ankle and typically walk with hindfoot valgus alignment. As the neutral weight-bearing axis of the lower limbs is restored with Total Knee Arthroplasty (TKA), ankle and hindfoot biomechanics also acutely change. This study aims to investigate whether any ankle clinical-radiographical changes occur as a result of bilateral mechanical TKA in patients with bilateral Osteoarthritis knee at a minimum follow-up of 6 months.

**Methods:**

The prospective observational study included 61 patients (122 knees) undergoing simultaneous bilateral TKA (mechanical alignment). Tibio-talar angle(TTA), tibial Anterior Surface angle (TAS), lateral distal tibial angle (LDTA), talar-tilt angle (TT), anatomical talocrural angle (aTC), ground surface and distal tibial plafond angle (GP), ground surface and an upper surface of talus angle (GT)and tibial plateau and tibial plafond angle (PP) were measured on long-film radiographs to look for changes in the ankle, whereas functional assessment was done using American Foot and Ankle Society (AOFAS), Foot and Ankle Disability Index (FADI), and Forgotten Joint (FJS-12) scores. Patients were sub-grouped based on the Hip-Knee-Ankle (HKA) axis, and the effect of the severity of knee varus on the ankles after TKA was also analyzed. The minimum follow-up was 6 months.

**Results:**

A significant decrease in the tibial plateau-tibial plafond (PP), ground-tibial plafond (GP), and ground-talar dome (GT) angles was noted after TKA (*p*-value < 0.05). Postoperative functional parameters were comparable to the preoperative status except for FADI, which significantly improved (*p*-value-0.03). Sub-group analysis based on the severity of knee varus (HKA) revealed GT to be most significantly reduced (*p*-value-0.036), while the talar tilt (TT) increased (*p*-value-0.044). Functional outcomes of the ankles clinically improved with the correction of severe knee varus after TKA. At a mean follow-up of 13.2 months post-TKA, 7 out of 61 (11.4%) patients complained of post-TKA ipsilateral ankle pain.

**Conclusion:**

Mechanically aligned bilateral TKA in severe varus deformity of the knee significantly decreases the GT angle but increases the varus tilt of the talus with lateral talar incongruency and under-coverage. Although the acute correction of severe knee varus deformity aligns the tibia more neutrally, resulting in an overall clinically evident improvement in ankle functional outcome, the increased varus talar tilt remains a deep concern.

**Level of Evidence:**

Prospective, observational, comparative study Level II.

## Introduction

The knee joint plays a major role in weight transmission of the body in day-to-day activities. The biomechanics of the knee and malalignment in lower limb anatomy are major risk factors for chronic knee pain, leaving Total Knee Arthroplasty (TKA) as the best and most successful option for patients with end-stage osteoarthritis (OA) [1–4]. As the neutral weight-bearing axis of the lower limbs is restored from its long-standing deformed state with TKA [5, 6], the ankle and hindfoot foot biomechanics also acutely alter [7, 8]. This can often lead to complaints of debilitating ankle pain after TKA [9].

Literature has reported very high percentages of patients almost as high as 24%-35% with concomitant ankle arthritis as with time, degenerative changes also develop in ankle joints among patients receiving TKA for their knee OA with severe knee varus deformities [10]. Some studies have even highlighted that ankle arthritis often progresses after TKA, and at 3 years follow-up 22% of patients with ankle pain were newly diagnosed with ankle OA because of increased valgus malalignment of the ankle [11–14]. However, a majority of the patients with ankle arthritis are due to varus or medial ankle tilt not being compensated at the subtalar joint with its valgus alignment [15–18].

Patients with varus knee osteoarthritis usually compensate at the ankle and walk with a hind foot valgus alignment as the medial arch of the foot gets more loaded [8]. This may be a reason for the chronic ankle functional changes in this subset of patients and there is a chance that the patient gets functional improvement in the ankle post-surgery [19, 20]. The present study aims to investigate any clinical-radiographical changes that occur at the ankle joint as a result of mechanically aligned bilateral TKA in patients with bilateral OA knee and whether patients experience improvement in their ankle functional outcomes post-surgery, with a minimum follow-up of 6 months. The null hypothesis of the study is that there will be no difference in clinical and radiographical outcomes before and after TKA.

## Methodology

**Fig. 1:**
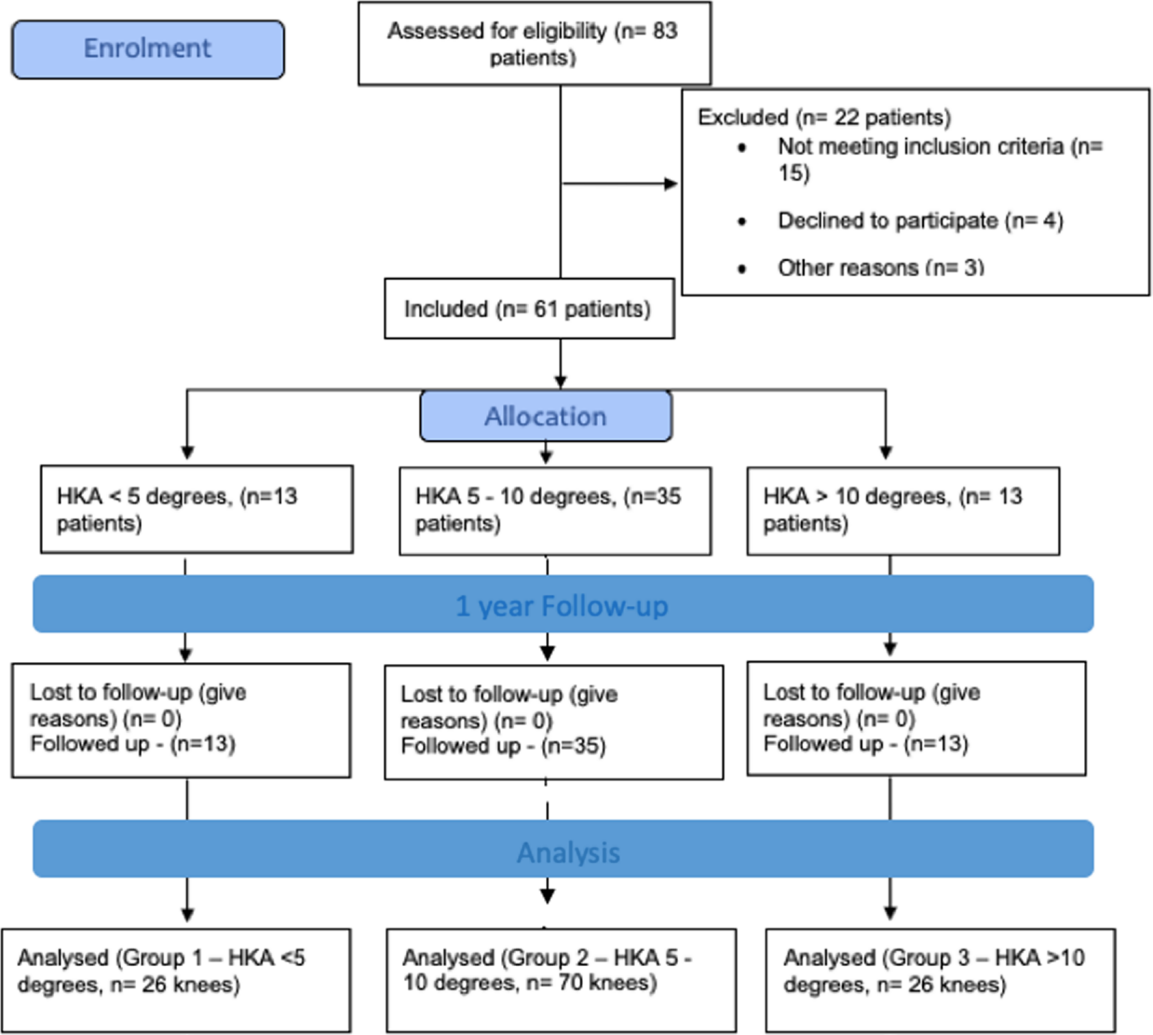
Flow diagram for patient recruitment

A prospective observational study was conducted among all patients undergoing sequential bilateral TKA in same sitting for end-stage OA knees from November 2020 to July 2022 at a tertiary care teaching institute. Written and informed consent were obtained from all participants, and ethical clearance was obtained from the independent institutional ethics committee. Sixty-one consecutive patients (122 knees) with primary end-stage knee OA and varus deformity undergoing simultaneous mechanically aligned bilateral TKA were included. Any patient receiving bilateral TKA for inflammatory knee arthritis or undergoing unilateral TKA, or with any history of septic arthritis of either knee or ankles and with any history of previous trauma or surgical intervention of their ankles or with any ankle deformity were excluded from this study. Patients undergoing revision knee arthroplasty or any case with associated spine-knee syndrome which may be affecting their overall functional status were also excluded (Fig. 1). The minimum follow-up was kept to be 12 months.

### Preoperative planning

**Fig. 2:**
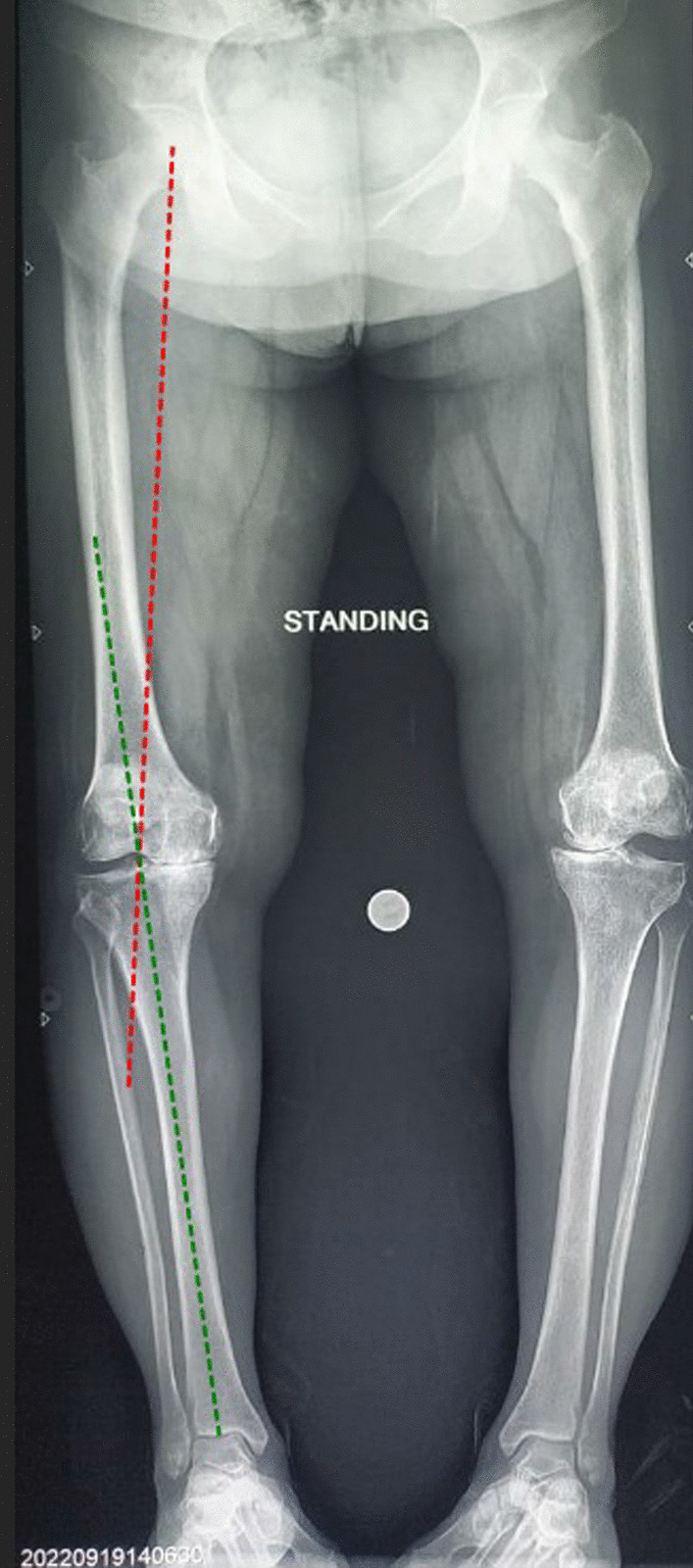
Hip-knee-ankle (HKA) Angle

Patients were planned on the preoperative long film weight-bearing plain radiographs, including the bilateral hip joints, knee joints, and ankles, using standard digital templating software (mediCAD Hectec GmbH, Germany) [21]. Hip-knee-ankle (HKA) angle were drawn to check the severity of knee varus (Fig. 2). Patients were divided into three groups based on their HKA angles, < 5 degrees (mild varus), 5–10 degrees (moderate varus), and > 10 degrees (severe varus).

### Surgical procedure

All patients underwent mechanically aligned bilateral TKAs using the standard medial parapatellar approach, under limited tourniquet use. Limb alignment was restored to the neutral mechanical alignment with an overall alignment complete correction of ± 3 degrees. The medial release was performed diligently in all cases, and if required in cases of significant medial tightness, a superficial medial collateral ligament needle pie crusting was considered. A single senior arthroplasty surgeon performed all the cases using either Attune or PFC sigma cemented posteriorly stabilized knee implants (DePuy Synthes, Warsaw, IN, USA) [22, 23]. A few cases were done using semi-constrained TC3 components (DePuy Synthes, Warsaw, IN, USA) where primary mediolateral balance was noted to be insufficient. Femoral and tibial intramedullary stems were used in cases where TC3 implants were required. Some cases with significant patella-femoral OA required an all-polyethylene patellar prosthesis for patellar resurfacing. In cases of clinically evident medial bone defects not amenable with only bone cement-prosthesis placement, either a screw with cement or bone autograft fixation method was used for posteromedial defect reconstruction. Patellar tracking was verified using the “no thumb technique” [24, 25] throughout the full knee flexion range of motion and lateral release was performed only if necessary.

### Postoperative rehabilitation

Postoperatively, all patients were rehabilitated following the same standard protocol. Since the first postoperative day, patients were started on full range of motion exercises within the limits of pain, rigorous quadriceps strengthening, and full weight-bearing mobilization using walking frames. Patients were discharged to their houses with a written and explained post-surgery rehabilitation protocol when they could ambulate independently out of the bed with walking frames with good quadriceps strength and no extension lags, and their pain was in control with oral analgesics, usually by the postoperative third day.

### Outcome measures

#### Radiographical outcomes

**Fig. 3:**
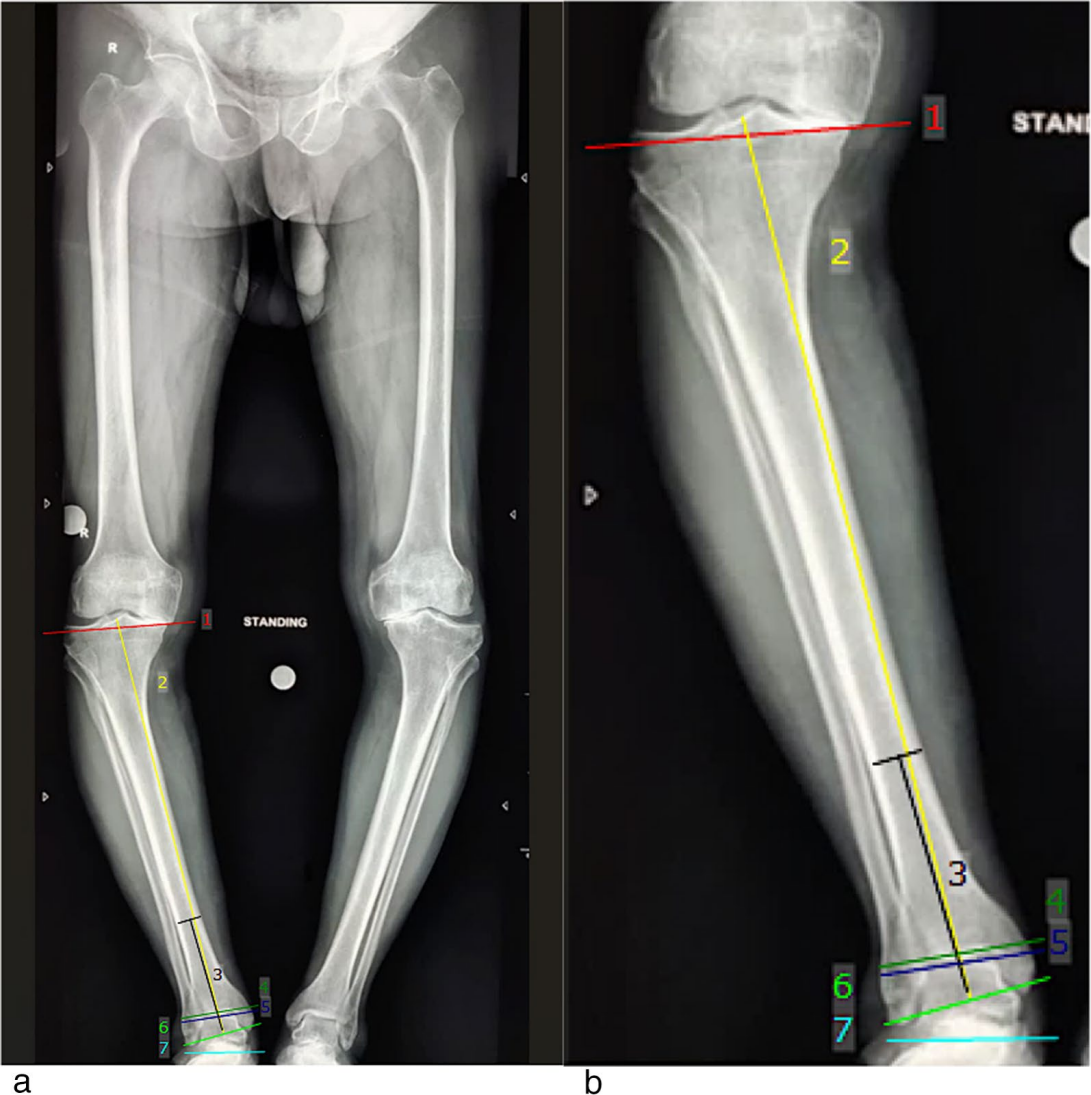
Pre-op Radiological parameters. The angle between: lines 1 and 4: Tibial Plateau and Tibial plafond angle, lines 3 and 5: Tibiotalar angle, lines 2 and 4: lateral distal tibial angle, lines 3 and 4: Tibial anterior surface angle, lines 4 and 5: Talar tilt, Lines 3 and 6: Talocrural angle, lines 4 and 7: Ground and distal tibial plafond angle, lines 5 and 7: Ground surface and upper surface of talus

**Fig. 4:**
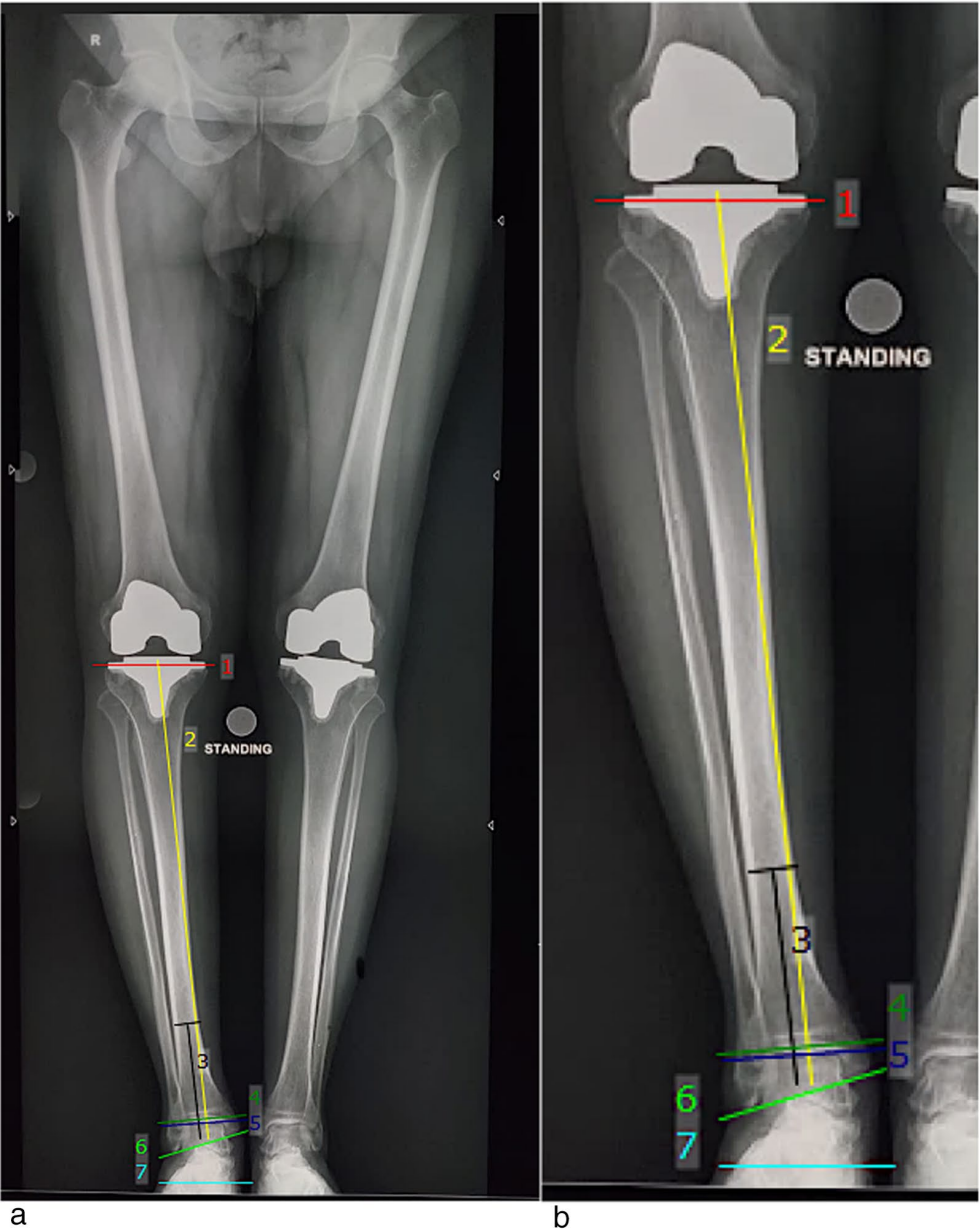
Post-op Radiological parameters. The angle between: lines 1 and 4: Tibial Plateau and Tibial plafond angle, lines 3 and 5: Tibiotalar angle, lines 2 and 4: Lateral distal tibial angle, lines 3 and 4: Tibial anterior surface angle, lines 4 and 5: Talar tilt, lines 3 and 6: Talocrural angle, lines 4 and 7: Ground and distal tibial plafond angle, lines 5 and 7: Ground surface and upper surface of talus

Radiographical ankle parameters were calculated on the standard long film plain radiographs of the bilateral lower limbs, both preoperatively and postoperatively at a minimum 1-year follow-up. Two independent orthopaedic trainee residents calculated the various ankle parameters on two separate occasions, 2 weeks apart (Figures 3 and 4).

1. Tibio-talar angle (TTA)—is defined as the angle subtended between the anatomical axis of the Tibia and the line drawn through the upper surface of the talus.
2. Tibial Anterior Surface angle (TAS)—the angle formed between the anatomical axis of the Tibia and the line drawn through the tibia plafond.
3. Lateral distal tibial angle (LDTA)—is defined as the angle between the two lines joining the centre of the tibial plateau to the centre of the ankle joint and distal tibial plafond.
4. Talar-Tilt angle (TT)—it is defined as the angle between the lines joining the distal tibial plafond and the upper surface of the talus.
5. Anatomical talocrural angle (aTC)—It is defined as the angle subtended between the line drawn through the anatomical axis of the tibia and the line joining the tip of the medial and lateral malleoli.
6. Ground surface and distal tibial plafond angle (GP)—It is defined as the angle subtended between the distal tibial plafond and the line drawn parallel to the ground surface.
7. Ground surface and an upper surface of talus angle (GT)—it is defined as the angle formed between the line drawn through the upper surface of the talus and another line drawn parallel to the ground surface.
8. Tibial plateau and tibial plafond angle (PP)—The angle formed between the line drawn through the tibial plateau and the line through the distal tibial plafond is the PP angle.

#### Clinical outcomes

Functional outcomes of the bilateral ankles among patients undergoing bilateral TKA were noted preoperatively and were compared with the postoperative scores at 1-year follow-up using valid clinical scoring systems. The American Foot and Ankle Society (AOFAS) score, foot and ankle disability index (FADI), and forgotten joint score (FJS-12) were used to determine and compare any ankle functional status changes after TKA.

### Statistical analysis

Continuous data are presented as means and standard deviations (SD), and categorical data have been represented in terms of absolute numbers and percentages. Categorical variables were compared using the chi-square test. All parameters were verified for their normal distribution using the Shapiro–Wilk Test. None of the continuous radiographical or clinical variables were noted to be non-parametric. Continuous parametric variables for pre-operative and post-operative data were compared using the paired T-test. A sub-group analysis was considered according to the mild, moderate, or severe degrees of varus based on the HKA angle, and the clinic-radiographical parameters were compared by two-way univariate ANOVA test, with post-hoc Tukey’s Honestly-Significant-Difference test to detect any difference between the groups. The Pearson correlation test was applied to check for any correlation between the changes in the radiographical and clinical functional scores of the ankles after TKA. Statistical tests employed for data analysis were always two-sided, with a level of significance set at five per cent. Results were considered statistically significant when the *p*-value was less than 0.05.

Intraobserver and interobserver reliability were tested by repeated measurements of all the radiographical parameters. The results will be expressed as intraclass correlation coefficients (ICC) with a 95% confidence interval (CI). All statistical analyses were conducted using SPSS software version 25.0 (SPSS Inc., Chicago, IL, USA). A post-hoc power analysis was conducted, and the current study was found to be adequately powered. The statistically significant ankle radiographical parameter being affected by correction of varus knee in the present study, GP (mean), and the GT values (mean, SD) suggested by a previous report by Lee et al. [16] were used, and the power of the study was detected to be 93.5%.

## Results

In this study, 61 consecutive patients (122 knees) with varus deformity who underwent bilateral mechanically aligned simultaneous TKA, achieving a neutral limb alignment were included and followed up postoperatively for at least a year (mean 13.2 months, SD 1.24, range 12–16.7 months). The baseline demographic details of all the patients are tabulated in Table 1. Preoperative and postoperative radiographical parameters were compared (Table 2) for all included patients. The PP, GP, and GT angles were noted to be significantly decreasing after bilateral TKA, whereas TT increased post-operatively (Table 2). Clinical outcomes were also compared, and all postoperative parameters were noted to be comparable to their preoperative functional status except FADI, which was noted to increase significantly after TKA (Table 3). There were 13 patients belonging to the mild varus (HKA < 5 degrees), 35 patients in the moderate varus (5–10 degrees), and 13 patients in the severe varus (> 10 degrees) group. Post-operatively, the HKA axis was noted to be 1.6, 0.98 degrees (mean, SD). Sub-group analysis was also conducted to determine the severity of varus knee affecting the various radiographical parameters, and only GT was noted to be significantly decreasing after surgery (Table 4). As the severity of knee varus increases, the GT tends to decrease largely after TKA from a comparatively high value, preoperatively. Sub-group analysis was also conducted between the various ankle functional scores. AOFAS, FADI, and FJS-12 were statistically comparable among the three groups of patients according to HKA angles (Table 5). In patients with HKA > 10 degrees, the ankle functional outcomes improved after TKA, clinically, but it was statistically insignificant. No correlation between the changes in the radiographical parameters and any improvement in functional ankle status after TKA was observed (Table 6). All radiological parameters were checked twice by two interpreters and good to excellent intraclass correlation coefficient was observed (Table 7).

**Table 1:** Baseline demographic details of all patients

Variables	Patient demographics
Age (years) (Mean, Range, SD)	65.66, 52–79, 3.47
Sex (male/females)	15/46
BMI [kgs/m^2^] (Mean, Range, SD)	32.1, 28.4–38.6, 1.27
HKA (mild, moderate, severe)	13, 35, 13 (21.3%, 57.4%, 21.3%)

**Table 2:** Comparison of pre-op and post-op radiological ankle parameters

Variables	Pre-operative radiological ankle parameters (mean, SD, 95%CI)	Post-operative radiological ankle parameters (mean, SD)	*P* value
Tibio-talar angle (TTA) [in degrees]	93.10, 3.67, 92.44–93.27	93.28, 4.77, 92.42–94.13	0.654^a^
Tibial Anterior Surface angle (TAS) [in degrees]	93.13, 3.52, 92.5–93.76	93.63, 3.95, 92.93, 94.34	0.141^a^
Lateral distal tibial angle (LDTA) [in degrees]	86.49, 7.79, 85.09–87.89	86.96, 3.5, 86.33–87.59	0.512^a^
Talar-Tilt angle (TT) [in degrees]	0.98, 0.89, 0.09–1.38	1.14, 1.14, 0.93–1.34	**0.020**^**a**^
Anatomical talo-crural angle (aTC) [in degrees]	84.6, 4.03, 83.88–85.32	84.09, 4.48, 83.29–84.89	0.248^a^
Tibial plateau and tibial plafond angle (PP) [in degrees]	4.53, 3.05, 3.9–5.08	3.37, 2.81, 3.25–4.25	**0.019**^**a**^
Ground surface and distal tibial plafond angle (GP) [in degrees]	5.48, 4.02, 4.7–6.20	2.92, 2.29, 2.51–3.33	**0.001**^**a**^
Ground surface and upper surface of talus angle (GT) [in degrees]	5.29, 4.06, 4.56–6.02	2.83, 2.33, 2.41–3.25	**0.001**^**a**^

*P*-value^a^—calculated using Paired *T* test, significant *P* value has been shown in bold type

**Table 3:** Comparison between pre-op and post-op clinical ankle parameters

Variables	Pre-operative (mean, SD, 95% CI)	Post-operative (mean, SD, 95% CI)	*P*-value
AOFAS	94.32, 6.79, 93.11–95.54	95.01, 5.95, 93.94–96.04	0.289^a^
FADI	95.42, 8.78, 93.85–97	96.22, 6.83, 95–97.45	**0.03**^**a**^
FJS-12 (ankles)	97.63, 8.23, 96.16–99.11	97.91, 6.29, 96.7–99.04	0.165^a^

*p*-value^a^—calculated using Paired *T* test, Significant *P* value has been shown in bold type

**Table 4:** Sub-group analysis and comparison of pre-op and post-op radiological ankle parameters

Variables	Pre-operative radiological ankle parameters (mean, SD, 95%CI)			Post-operative radiological ankle parameters (mean, SD)			*P* value
	HKA < 5	HKA 5–10	HKA > 10	HKA < 5	HKA 5–10	HKA > 10	
Tibio-talar angle (TTA) [in degrees]	93.30, 3.25, 91.98–94.61	93.11, 3.43, 91.73–94.50	91.92, 2.91, 90.74–93.1	92.68, 4.97, 90.68–94.69	92.27, 4.64, 90.39–94.14	92.58, 3.91, 91.0–94.16	0.611^a^
Tibial Anterior Surface angle (TAS) [in degrees]	93.35, 2.45, 92.35–94.34	93.45, 2.37, 92.5–94.41	92.29, 2.58, 92.35–94.34	93.65, 3.43, 92.26–95.04	93.32, 3.49, 91.19–94.73	91.98, 3.21, 90.68–93.28	0.338^a^
Lateral distal tibial angle (LDTA) [in degrees]	87.69, 2.27, 86.77–88.61	87.43, 2.68, 86.34–88.52	88.46, 2.25, 87.55–89.38	87.55, 3.27, 86.23–88.88	87.12, 3.2, 85.82–88.43	87.86, 2.86, 86.7–89.02	0.163^a^
Talar-Tilt angle (TT) [in degrees]	0.89, 0.77, 0.57–1.2	0.78, 0.622, 0.53–1.03	0.8077, 0.57, 0.577–1.03	0.82, 0.719, 0.53–1.1	1.3, 0.75, 1.00–1.6	1.52, 1.96, 0.73–2.32	**0.044**^**a**^
Anatomical talo-crural angle (aTC) [in degrees]	84.88, 2.45, 83.89–85.87	84.62, 4.69, 82.72–86.52	84.05, 4.33, 82.3–85.8	83.68, 3.95, 82.03–85.22	84.05, 5.24, 81.94–86.17	84.47, 4.73, 82.56–86.38	0.617^a^
Tibial plateau and tibial plafond angle (PP) [in degrees]	4.18, 3.02, 2.9–5.4	3.45, 2.28, 2.5–4.3	6.49, 3.29, 5.1–7.8	3.6, 2.84, 2.4–4.75	3.6, 225, 2.2–4.57	2.83, 2.72, 1.7–3.93	0.806^a^
Ground surface and distal tibial plafond angle (GP) [in degrees]	5.83, 4.09, 4.17–7.48	4.2, 2.57, 3.16–5.24	9.15, 3.66, 7.6–10.62	2.78, 2.23, 1.8–3.68	2.86, 1.94, 2.08–3.65	2.71, 1.84, 1.9–3.46	0.828^a^
Ground surface and upper surface of talus angle (GT) [in degrees]	5.4, 4.18, 3.7–7.16	3.91, 2.2, 3.017–4.81	9.3, 3.83, 7.7–10.86	2.65, 2.23, 1.7–3.55	2.9, 1.92, 2.12–3.68	2.8, 2.24, 1.9–3.7	**0.036**^**a**^

*P*-value^a^—calculated by two-way Anova test, where the dependent variable HKA angle shown to be affecting the fixed pre-operative and post-operative parameters, Significant *P* value has been shown in bold type

**Table 5:** Sub-group analysis and comparison of pre-op and post-op clinical ankle parameters

Variables	Pre-operative radiological ankle parameters (mean, SD, 95% CI)			Post-operative radiological ankle parameters (mean, SD)			*P* value
Groups	HKA < 5	HKA 5–10	HKA > 10	HKA < 5	HKA 5–10	HKA > 10	
AOFAS	97.15, 3.49, 95.74–98.56	95.07, 5.52, 92.84–97.3	90.88, 10.12, 86.79–94.97	96.5, 3.73, 95.03–98.04	95.84, 4.92, 93.85–97.83	94.23, 6.5, 91.6–96.85	0.487^a^
FADI	97.87, 3.75, 96.35–99.38	95.21, 5.53, 92.98–97.45	92.63, 16.12, 86.1–99.14	97.29, 4.66, 95.4–99.17	95.95, 4.57, 94.1–97.8	94.57, 11.3, 89.99–99.15	0.982^a^
FJS-12 (ankles)	99.56, 1.32, 99.02–100	98.97, 1.83, 98.95–99.16	92.53, 16.79, 85.7–99.31	99.48, 1.36, 98.93–100.03	98.95, 1.83, 98.2–99.69	93.84, 12.5, 88.7–98.91	0.992^a^

*P*-value^a^—calculated by two-way Anova test, where the dependent variable HKA angle shown to be affecting the fixed pre-operative and post-operative parameters

AOFAS—American Orthopaedic Foot and ankle society, FADI—Foot and Ankle Disability Index, FJS-12—Forgotten Joint Score—12, HKA- Hip Knee Ankle Angle

**Table 6:** Correlation between clinical and the changes between radiological ankle parameters after Bilateral TKA

Variables	Radiological ankle parameters							
	TTA	TAS	LDTA	TT	aTC	PP	GP	GT
AOFAS	Pearson Correlation	− 0.036	− 0.019	− .0037	0.054	0.014	− 0.001	− 0.049
	Sig. (2-tailed)	0.577	0.770	0.572	0.409	0.829	0.986	0.451
FADI	Pearson Correlation	− 0.133	− 0.106	0.070	0.004	− 0.083	− 0.078	− 0.075
	Sig. (2-tailed)	0.19	0.100	0.282	0.950	0.202	0.231	0.245
FJS-12	Pearson Correlation	0.058	0.063	− 0.049	0.021	0.026	− 0.033	− 0.132
	Sig. (2-tailed)	0.367	0.329	0.446	0.746	0.692	0.611	0.421

AOFAS—American Orthopaedic Foot and ankle society, FADI—Foot and Ankle Disability Index, FJS-12—Forgotten Joint Score—12

**Table 7:** Intra and interobserver reliability comparison between pre-op and post-op radiological ankle parameters

Variables	Pre-operative radiological ankle parameters		Post-operative radiological ankle parameters	
	Intra-observer reliability (ICC, 95% CI)	Inter-observer reliability (ICC, 95% CI)	Intra-observer reliability (ICC, 95% CI)	Inter-observer reliability (ICC, 95% CI)
Tibio-talar angle (TTA)	0.987^a^ (0.952–0.991)	0.948 (0.885–0.977)	0.946^a^ (0.903–0.980)	0.925 (0.837–0.966)
	0.925^b^ (0.880–0.975)		0.965^b^ (0.908–0.981)	
Tibial Anterior Surface angle (TAS)	0.921^a^ (0.845–0.968)	0.967 (0.916–0.983)	0.910^a^ (0.890–0.978)	0.889 (0.864–0.972)
	0.934^b^ (0.920–0.984)		0.973^b^ (0.918–0.983)	
Lateral distal tibial angle (LDTA)	0.823^a^ (0.794–0.956)	0.937 (0.905–0.981)	0.921^a^ (0.891–0.978)	0.878 (0.846–0.968)
	0.789^b^ (0.646–0.920)		0.915^b^ (0.817–0.962)	
Talar-Tilt angle (TT)	0.929^a^ (0.846–0.968)	0.930 (0.827–0.964)	0.824^a^ (0.779–0.95)	0.932 (0.852–0.969)
	0.954^b^ (0.900 -0.980)		0.934^b^ (0.829–0.962)	
Anatomical talo-crural angle (aTC)	0.867^a^ (0.745–0.945)	0.814 (0.774–0.952)	0.917^a^ (0.808–0.960)	0.942 (0.888–0.977)
	0.92^b^ (0.808–0.960)		0.924^b^ (0.889–0.977)	
Ground surface and distal tibial plafond angle (GP)	0.926^a^ (0.845–0.968)	0.962 (0.916–0.983)	0.951^a^ (0.890–0.978)	0.924 (0.864–0.972)
	0.924^b^ (0.880–0.975)		0.958^b^ (0.908–0.981)	
Ground surface and upper surface of talus angle (GT)	0.924^a^ (0.794–0.956)	0.927 (0.905–0.981)	0.951^a^ (0.891–0.978)	0.938 (0.864–0.972)
	0.817^b^ (0.646–0.920)		0.917^b^ (0.817–0.962)	
Tibial plateau and tibial plafond angle (PP)	0.919^a^ (0.846–0.968)	0.92 (0.827–0.964)	0.812^a^ (0.732–0.953)	0.929 (0.846–0.968)
	0.928^b^ (0.900–0.980)		0.915^b^ (0.839–0.925)	

Intra-observer reliability ICC^a^— Between observer 1 at two separate occasions 2 weeks apart, Intra-observer reliability ICC^b^—Between observer 2 at two separate occasions 2 weeks apart

In our cohort, 7 out of 61 (11.4%) patients complained of post-TKA ipsilateral ankle pain, although the ankle functional outcomes, joint perception, and awareness improved after TKA (Table 5).

## Discussion

The present study highlights that out of all eight coronal ankle radiological alignment parameters affecting the radiographical ankle outcomes after a mechanically aligned bilateral TKA, the PP, GT, and GP angles were significantly affected. Sub-group analysis according to the severity of knee varus revealed that, of these radiographical parameters, the GT angle is the most affected parameter which corrects, especially among patients with severe varus deformity at the knee (HKA > 10). The tibiotalar tilt angle increases from its preoperative measures after the acute correction of severe knee varus deformity with TKA. Although the talus tilts medially with the tibia aligning more neutrally, resulting in lateral talar incongruencies in severe knee varus deformity patients, it leads to an overall clinically evident improvement in ankle functional outcomes after surgery. Clinically the FADI outcome score improved significantly post TKA suggesting better ankle functionality and patient satisfaction.

Despite TKA being the most successful option for end-stage knee osteoarthritis, almost 20% of patients remain dissatisfied after surgery. There may be a causal relationship between the change in ankle alignment post-surgery, which might be affecting their post-surgery satisfaction level. OA is one of the most debilitating diseases of the older age group and a major cause of TKA around the world, and often a high percentage of patients have concomitant asymptomatic ankle OA. OA knee results in a significant reduction in working efficiency and causes a great amount of morbidity to the patient. TKA can alleviate knee pain and enhance knee range of motion, but it may exacerbate ankle pain. Due to more medial shifting of the weight-bearing axis beyond the medial knee compartment as the severity of varus knee deformity increases, compensatory anatomical deformities at the ankle joint and hindfoot valgus alignment develop.

Mullaji et al. [19] was the first to incorporate the hindfoot valgus state in patients undergoing TKA for varus deformity. They suggested that the preoperative hind foot valgus decreased after TKA with the restoration of the limb neutral alignment, but there remains a persistent valgus deformity which leads to the ground mechanical axis passing laterally to the knee. Even Kapoor et al. [26] suggested in their study that the preoperative valgus of the hindfoot decreases after TKA, and this may be an indirect effect of overall functional improvement in the hindfoot’s functional status. The present prospective study also highlights a similar improvement in functional status after TKA. Xie et al. [27] described the various hindfoot radiological parameters and these were studied in the present study to determine the hindfoot alignment. The radiological parameters in the current study also highlight a varus incongruency or varus exaggeration of the hindfoot, the more the knee varus deformity is corrected to a neutral mechanical alignment. Feng et al. [28] in their systematic review highlights the cause of pain to be because of residual varus alignment of the ankle with a stiff hindfoot or ankle arthritis. Lee et al. [29] postulated that overall valgus alignment leads to increased valgus-related hindfoot arthritis which was contrary to our findings, where we found a varus or a medially tilted hindfoot after correction of severe knee varus deformity. Chang et al. [11] reports a similar study finding in their study.

**Fig. 5:**
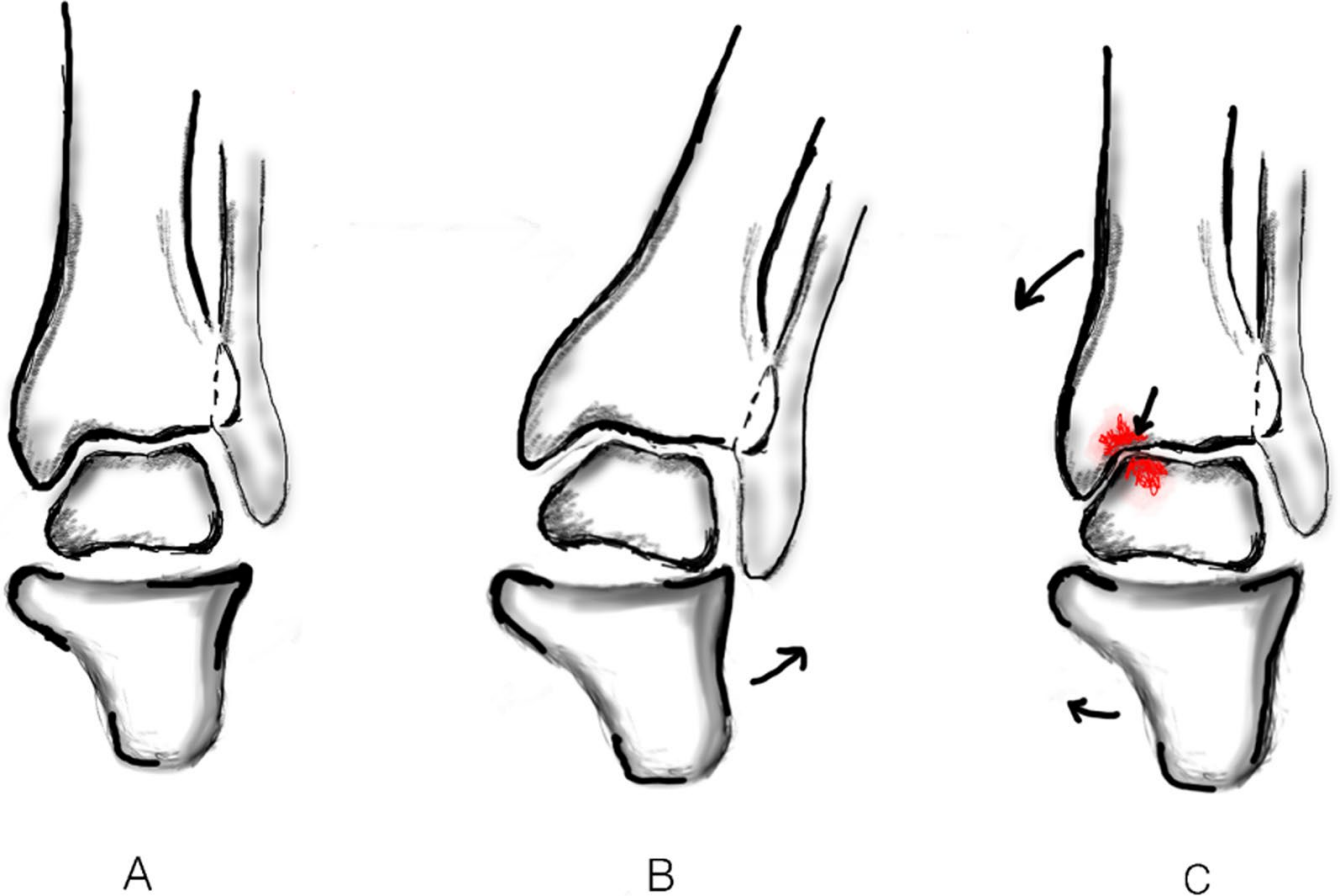
Ankle loading in knee varus. **A** Normal alignment of the hindfoot, **B** with the development of varus knee deformity, hindfoot goes into valgus, **C** correction of Varus knee deformity, reduces the hindfoot valgus but increases medial talar tilt and medial load-shifting (shown in Red). The outward arrow in **B** indicates the compensatory hindfoot valgus with development of knee varus. The inward arrow in **C** indicates the correction of tibia to its neutral alignment after mechanically aligned TKA and the decrease in hindfoot valgus

Upon further analysis of the current study findings, it suggested that the TT angle increases after TKA (Table 2) similar to the finding of Chang et al. [11] as a more severe varus knee deformity is acutely corrected to a neutral position according to mechanical alignment philosophy. This can be explained by the fact that as the tibia corrects to a neutral coronal position, the talar position remains unchanged even though the supple subtalar joint compensating for the constitutional hindfoot valgus reduces. Previous studies have already established the compensatory hindfoot valgus reduces with severe varus knee deformity correction in TKA [26, 30–33]. Possibly, the subtalar valgus reduction affects the tibio-talar joint by reducing the talar varus tilt which is evident through the decrease in the GT and GP angles as the severity of knee varus is corrected (Tables 2, 3). This is a biomechanically advantageous phenomenon for the hindfoot as it helps in the overall improvement of the hindfoot’s functional status, ankle joint perception, and awareness (Table 3). However, the compensatory effect of the reduction in hindfoot valgus, thereby affecting the reduction in the talar medial tilt, is possible only up to a certain limit. Beyond this, the tibial neutral correction becomes dominant leaving a residual medial talar tilt. Thus, the varus or medial tilt of the talus increases, with the neutral tibial positional changes resulting in more lateral talar incongruencies and under-coverage, leading to the medial load-shift over the talus (Fig. 5). The present study findings prove that with severe varus knee deformity correction by TKA, the PP angle decreases but the TT angle increases. This is a probable cause of the generation of ankle pain in some patients and can predispose medial ankle arthritis after TKA in the long term. Under-correcting the native tibial varus according to functional alignment-TKA protocol can thus be beneficial for the patient as it will reduce the medial talar tilt, and this remains a future directive for further research.

The present study however comes with its own set of limitations. The significant radiologic changes seen in this study may be related to ankle OA in the long-term follow-up, but since the present report remains a short term study, this is an important limitation. A small sample size can also be considered as another drawback of the present study. However, the prospective study design and analysis of the ankle functional status after TKA remain, the possible strength. To the best of our knowledge, no previous articles have studied the ankle functional as well as radiological outcome changes after TKA. All radiological parameters were studied twice by two different independent observers on two separate occasions with good to excellent intra-class correlation coefficient, to avoid any possible discrepancies but still, the findings are observer-dependent which can also be indicated as a limitation. The knee varus deformities were only calculated by anteroposterior imaging, but the simultaneous presence of any sagittal flexion deformity which can underestimate the coronal varus deformity was also out of the scope of this present study. Long-term, multicentric studies to validate the current findings are required to complement the present study results.

## Conclusion

Mechanically aligned bilateral TKA in severe varus deformity of the knee significantly decreases the GT angle but increases the varus tilt of the talus, resulting in lateral talar incongruency and under-coverage. Although the acute correction of severe knee varus deformity aligns the tibia more neutrally, leading to an overall clinically evident improvement in ankle functional outcome, the increased varus talar tilt remains a significant concern.

## Data Availability

All the data was made available after direct observation.
